# High Expression of CXCL10/CXCR3 in Ventilator-Induced Lung Injury Caused by High Mechanical Power

**DOI:** 10.1155/2022/6803154

**Published:** 2022-01-07

**Authors:** Yongpeng Xie, Hui Zheng, Zhifang Mou, Yanli Wang, Xiaomin Li

**Affiliations:** ^1^Department of Critical Care Medicine, Lianyungang Clinical College of Nanjing Medical University, The First People's Hospital of Lianyungang, Lianyungang, China; ^2^Department of Emergency Medicine, Lianyungang Clinical College of Nanjing Medical University, The First People's Hospital of Lianyungang, Lianyungang, China

## Abstract

**Background:**

The energy delivered by a ventilator to the respiratory system in one minute is defined as mechanical power (MP). However, the effect of ventilator-induced lung injury (VILI) in patients suffering from acute respiratory distress syndrome (ARDS) is still unknown. Our previous studies revealed that CXCL10 may be a potential biomarker of lung injury in ARDS. Therefore, the aim of this study was to compare the lung injury of rats and patients under different MP conditions to explore the involvement of CXCL10 and its receptor CXCR3 in VILI.

**Methods:**

Patients were divided into the high mechanical power group (HMPp group) and low mechanical power group (LMPp group), while rats were assigned to the high mechanical power group (HMPr group), medium mechanical power group (MMPr group), and low mechanical power group (LMPr group). CXCL10 and CXCR3 plasma content in ARDS patients and rats under ventilation at different MP was measured, as well as their protein and mRNA expression in rat lungs.

**Results:**

CXCL10 and CXCR3 content in the plasma of ARDS patients in the HMPp was significantly higher than that in the LMPp. The increase of MP during mechanical ventilation in the rats gradually increased lung damage, and CXCL10 and CXCR3 levels in rat plasma gradually increased with the increase of MP. CXCL10 and CXCR3 protein and mRNA expression in the HMPr group and MMPr group was significantly higher than that in the LMPr group (*P* < 0.05). More mast cells were present in the trachea, bronchus, blood vessels, and lymphatic system in the rat lungs of the HMPr group, and the number of mast cells in the HMPr group (13.32 ± 3.27) was significantly higher than that in the LMPr group (3.25 ± 0.29) (*P* < 0.05).

**Conclusion:**

The higher the MP, the more severe the lung injury, and the higher the CXCL10/CXCR3 expression. Therefore, CXCL10/CXCR3 might participate in VILI by mediating mast cell chemotaxis.

## 1. Introduction

Acute respiratory distress syndrome (ARDS) is one of the most common critical illnesses in intensive care medicine. An international epidemiological study (LUNG SAFE) reported that the ARDS incidence in the ICU is approximately 10% and its fatality rate is from 35% to 46% [1]. Mechanical ventilation is required in the treatment of ARDS, but improper use can also cause or aggravate lung injury, leading to pulmonary fibrosis and ventilator-induced lung injury (VILI), which is highly affecting the prognosis of critically ill patients [2]. The mortality rate of severe ARDS is as high as 40%, and many high-mortality factors are caused by the improper use of ventilators [3, 4]. VILI is the result of the combined effects of pressure, volume, flow rate, frequency, and other factors. However, any mechanical parameter used alone to assess the severity of ARDS does not reflect the effect of VILI. The concept of mechanical power (MP) combines the comprehensive effects of the above respiratory mechanical parameters; thus, it can allow a better evaluation and prevention of VILI during mechanical ventilation in patients with ARDS. Thus, it may become a new standard for guiding safe lung ventilation in ARDS patients. However, the injury can be caused by the high energy when the mechanical power is too high, becoming a direct pathogenic factor for VILI [5]. The overloaded energy inevitably leads to biological effects in the lungs, and it can be called energy-biological injury. However, it is not yet clear which signaling pathways or effect molecules are involved in the energy-biological injury during mechanical ventilation in ARDS patients. Therefore, according to our previous work [6], the role of mast cell chemotaxis mediated by CXCL10 and its receptor CXCR3 was further explored *in vivo* on patients and animal experiments.

## 2. Research Objects and Methods

### 2.1. Clinical Research

Eighty-five ARDS patients in the ICU ward of the First People's Hospital of Lianyungang who needed invasive mechanical ventilation were selected in sequence from June 2019 to May 2021, which included 55 males and 30 females. Their basic information including the APACHE II score and SOFA score were recorded in detail. All the enrolled patients met the 2012 ARDS Berlin-defined diagnostic criteria [7]. All the included patients were ventilated according to the original ARDSnet protocol [8]. Briefly, patients were ventilated in a volume-assisted control mode with a constant square flow and a tidal volume of 6 mL/kg/IPBW (ideal predicted body weight) using the PB840 ventilator (Tyco Healthcare USA). There would be a short pause to obtain the plateau pressure. The goal of oxygenation was to target a peripheral saturation of blood oxygen measured by pulse oximetry between 88 and 95% or a PaO_2_ of 55–80 mmHg measured by arterial blood gas analysis. To achieve this goal, FiO_2_ and PEEP were adjusted according to the table of PEEP and FIO_2_ combinations as in the ARMA study and ACURASYS study (Table 1) [9]. RR was adjusted to ensure an arterial pH between 7.20 and 7.45. The respiratory mechanical parameters were monitored at 0 h, 6 h, 18 h, and 24 h of the mechanical ventilation treatment and included the driving pressure, respiratory rate, flow rate, peak airway pressure, plateau airway pressure, tidal volume, positive end-expiratory pressure, and lung compliance. The mechanical power was calculated according to the classical simplified formula MP = 0.098 × RR × VT × (Ppeak − 1/2DP) [10, 11], in which RR is the respiratory rate, VT is the tidal volume, Ppeak is the peak airway pressure, and DP is the driving pressure, and the unit is J/min. The average of the measured values at four time points was considered the 24 h average MP. In addition, 5 mL blood samples were collected at 24 h after admission, and the serum was separated by centrifugation after allowing the sample to rest for 30 min and stored at -80°C for further use. The content of CXCL10 and CXCR3 in the serum was detected by enzyme-linked immunosorbent assay (ELISA). All patients were divided into the high mechanical power group (HMPp group) and low mechanical power group (LMPp group) according to the value of 24 h-mean-MP higher or lower than 17.0 J/min [10], and the differences in CXCL10 and CXCR3 between the two groups of patients were compared.

### 2.2. Animal Experiment

#### 2.2.1. Establishment of the Rat VILI Model

Twenty-four healthy and clean SD rats, 6-8 weeks old, weighing 240-260 grams, were used. They were divided into the control group (N group), low mechanical power group (LMPr group), medium mechanical power group (MMPr group), and high mechanical power group (HMPr group) according to the random number table, with 6 animals in each group. Tracheotomy was performed, and a tracheal tube was inserted and fixed. Rats in the N group underwent tracheotomy but were not subjected to mechanical ventilation. The LMPr group, MMPr group, and HMPr group received 60 mJ/min, 120 mJ/min, and 180 mJ/min mechanical ventilation [12], respectively (CWE, Inc., SAR-1000, USA). All the rats were anesthetized with intraperitoneal injections of xylazine (8 mg/kg) and ketamine (80 mg/kg). The other ventilator parameter standards were set as follows: tidal volume 8 mL/kg, inspiratory-expiratory ratio 1 : 1, positive end-expiratory pressure 3 cm H_2_O, and oxygen concentration (FiO_2_) 40%, so that each group of MP, respectively, reaches the level of 60 mJ/min, 120 mJ/min, and 180 mJ/min by adjusting the ventilator frequency. After 24 h of mechanical ventilation, the rats were sacrificed by cardiac puncture and the removal of the whole blood, and lung specimens were collected for examination.

#### 2.2.2. Pathological Damage in the Lung Tissue and Chemotaxis of Mast Cells in Rats

The tissue of the upper lobe of the right lung of the rats was collected, dehydrated by alcohol, embedded in paraffin, and sectioned. The pathological damage of the lung tissue was observed. The mast cells in the rat lung tissues were stained with toluidine blue, morphologically analyzed, and counted by evaluating the number of mast cells in 10 high-power fields (×400). The average number represented the density of mast cells, and the overall distribution in the trachea, bronchus, blood vessels, and lymphatic vessels was evaluated. Mast cells under normal conditions appear purple under the microscope after toluidine blue staining, and the background is light blue or colorless.

#### 2.2.3. Determination of the Wet/Dry Weight Ratio of the Lung Tissue

The tissue of the middle lobe of the right lung of the rats was collected, the wet weight was measured, and the sample was placed in an oven under a constant temperature of 75°C for 24 h until the tissue had a constant weight. Then, the dry weight of the tissue was measured, and the wet/dry weight ratio was calculated to determine the degree of pulmonary edema.

#### 2.2.4. Serum Inflammatory Factor by ELISA

ELISA was used to detect the levels of CXCL10, CXCR3, interleukin-10 (IL-10), and tumor necrosis factor-*α* (TNF-*α*) in the rat serum according to the manufacturer's protocol (Wuhan Yunclone Technology Co., Ltd.). The multifunctional enzyme immunoassay analyzer (BioTek Epoch, USA) was used for detection.

#### 2.2.5. CXCL10 and CXCR3 Protein Expression in the Lung Tissue by Western Blotting

The lower lobe of the right lung of the rats was collected and used for Western blotting to detect CXCL10 and CXCR3 protein expression in the lung tissue. GAPDH was used as the loading control to calculate the relative protein content of CXCL10 and CXCR3.

#### 2.2.6. CXCL10 and CXCR3 mRNA Expression in the Lung Tissue by RT-qPCR

The TRIzol® reagent (Invitrogen; Thermo Fisher Scientific, Inc.) was used to extract the total RNA from 100 mg left lung tissue of the rats, which was reverse transcribed into cDNA. The expression of CXCL10 and CXCR3 mRNA was measured by RT-qPCR using an Exicycler™ 96 real-time system (Bioneer) with SYBR Premix Ex Taq II (Takara Bio, Inc.). The PCR conditions were as follows: enzyme activation at 95°C for 5 min, amplification at 95°C for 15 sec, annealing at 56°C for 30 sec, and extension at 72°C for 20 sec, for a total of 44 cycles. Finally, the melting curve was performed (75°C → 95°C, heating up 1°C every 20 sec). The expression of the target genes CXCL10 and CXCR3 was quantified using the 2-*ΔΔ*Ct method. rGAPDH was used as the reference gene. The primers used were the following: GAPDH forward, 5′-CAAGTTCAACGGCACAGTCAAG-3′ and reverse, 5′-ACATACTCAGCACCAGCATCAC-3′; CXCL10 forward, 5′-GATGACGGGCCAGTGAGAAT-3′ and reverse, 5′-CTCAACACGTGGGCAGGATA-3′; and CXCR3 forward, 5′-GCTCTTTGCCCTCCCAGATT-3′ and reverse, 5′-AAGGGGCATCAGGAAACCAG-3′.

### 2.3. Ethics Approval and Consent to Participate

The research was approved by the ethics committee of the Lianyungang Clinical College of Nanjing Medical University with the approval number LCYJ20170312001. A written informed consent was obtained from the patient's legal representatives before the beginning of the study. Patient records/information was anonymous analysis. The registration number of this project in the China Clinical Trial Registration Center was ChiCTR1900028238. All methods were performed in accordance with the relevant guidelines and regulations, and this study is reported in accordance with ARRIVE guidelines.

### 2.4. Statistical Analysis

Statistical analysis was performed using the SPSS 22.0 statistical software and GraphPad Prism 6.0. Data were represented as *x* ± *s*, and the difference between the two groups was evaluated using the two independent sample *t*-tests. The *χ*^2^ test was used to compare the count data. A value of *P* < 0.05 was considered statistically significant.

## 3. Results

### 3.1. General Clinical Data of ARDS Patients

No statistical difference was found in gender, age, weight, body mass index, basic medical history (including hypertension, diabetes, coronary heart disease, and history of trauma and surgery), and smoking history between the HMPp group (46 cases) and the LMPp group (39 cases). However, APACHE II, SOFA, PaO_2_/FiO_2_, and blood lactic acid were statistically increased/decreased in the HMPp group compared to their values in the LMPp group (all *P* < 0.05, Table 1).

### 3.2. CXCL10 and CXCR3 Expression in the Two Groups of ARDS Patients

The serum levels of CXCL10 and CXCR3 in the HMPp group were significantly higher than those in the LMPp group, and the difference was statistically significant (all *P* < 0.05, Table 2).

### 3.3. Pathological Changes in the Rat Lung Tissue

**Figure 1:**
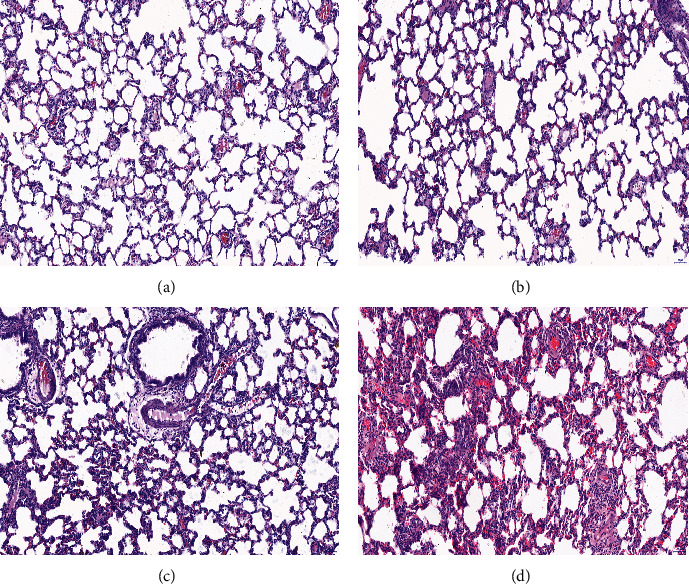
Pathological changes in the rat lung tissue under different levels of MP (HE, ×200).

The pathological changes in the lung tissues were observed after 24 hours of mechanical ventilation with different MP conditions, and no evident pathological changes in the lung tissues were found in the N group and LMPr group under the light microscope (Figures 1(a) and 1(b)). However, with the increase in MP during mechanical ventilation, that is, going from the MMPr group to the HMPr group, the pathological damage in the rat lung tissue gradually increased, as revealed by several features, including the diffuse alveolar injury, congestion, and edema in the alveolar cavity and interstitium, inflammatory cell infiltration, no stretching of the lung, and transparent film formation (Figures 1(c) and 1(d)).

### 3.4. Wet/Dry Weight Ratio of the Rat Lung Tissue

**Figure 2:**
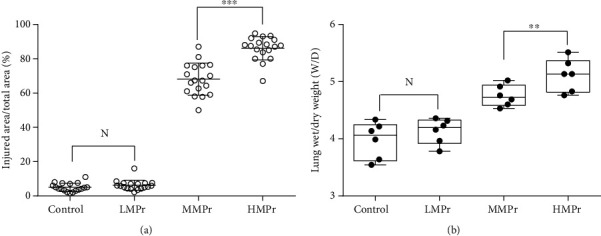
Analysis of the wet/dry weight ratio of the lung tissue.

The wet/dry weight ratio test of the lung tissue reflects the permeability of the pulmonary blood vessels, and its value can indicate the severity of pulmonary edema. After 24 h of mechanical ventilation, the wet/dry weight ratio of the lung tissue in the HMPr group was significantly higher than that in the LMPr group (5.253 ± 0.721 vs. 4.354 ± 0.246) (*P* < 0.01, Figure 2).

### 3.5. Number and Chemotaxis of Mast Cells in the Rat Lung Tissue

**Figure 3:**
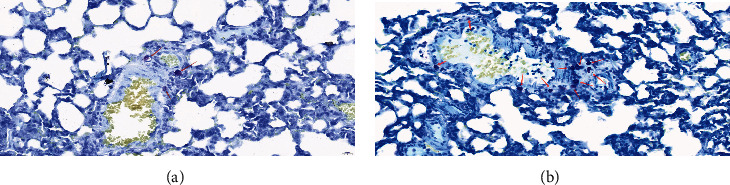
Chemotaxis and number of mast cells in the rat lung tissue under different levels of MP (TBS ×400).

More mast cells were found in the lung tissue of the rats in the HMPr group, distributed along the trachea, bronchus, blood vessels, and lymphatic vessels. The morphology of the mast cells was relatively regular, and the cytoplasm was rich in thick purple-red metachromatic particles, densely packed, sometimes occupying the entire cell. The nucleus was light blue or brightly located in the center of the cell or on one side. The number of mast cells in the HMPr group (13.323 ± 3.272) was significantly higher than that in the LMPr group (3.254 ± 0.295), and the difference was statistically significant (*P* < 0.01, Figure 3).

### 3.6. Level of CXCL10/CXCR3 and Other Cytokines in the Serum of Rats with Lung Injury

**Figure 4:**
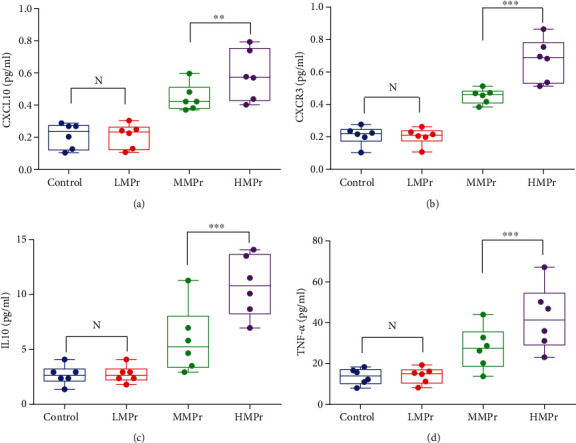
Inflammatory cytokine in the serum under different levels of MP.

The continuous increase of MP corresponded to a gradual aggravation of the lung injury. In addition, the serum levels of IL-10, TNF-*α*, CXCL10, and CXCR3 gradually increased in the HMPr group and were significantly higher than their levels in the N group (0.586 ± 0.064 vs. 0.210 ± 0.032, 0.674 ± 0.054 vs. 0.209 ± 0.023, 10.801 ± 1.132 vs. 2.674 ± 0.367, and 42.291 ± 6.418 vs. 13.662 ± 1.593, pg/mL) (*P* < 0.01) (Figure 4).

### 3.7. CXCL10 and CXCR3 Protein Expression in the Rat Lung Tissue

**Figure 5:**
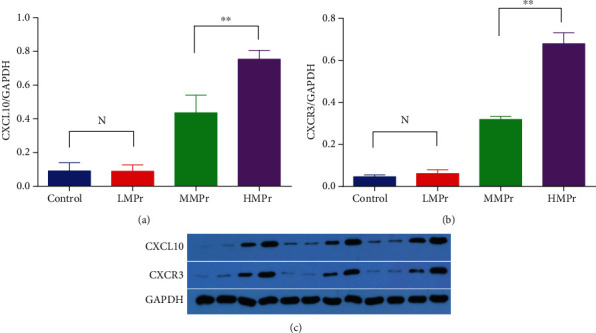
CXCL10/CXCR3 protein expression under different levels of MP.

CXCL10 and CXCR3 protein expression in the HMPr group and MMPr group after 24 hours of mechanical ventilation was significantly higher than that in the LMPr group (0.754 ± 0.030 vs. 0.090 ± 0.022, 0.679 ± 0.027 vs. 0.061 ± 0.010) (*P* < 0.01). No statistically significant difference was found in their expression between the LMPr group and the control group (Figure 5).

### 3.8. CXCL10 and CXCR3 mRNA Expression in the Rat Lung Tissue

**Figure 6:**
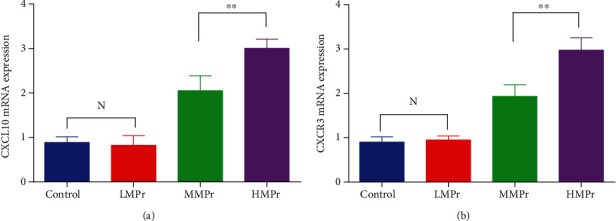
CXCL10/CXCR3 gene expression under different levels of MP.

The function of CXCL10 is mediated by its receptor CXCR3. The results showed that CXCL10 and CXCR3 mRNA expression in the HMPr group was significantly upregulated compared with their expression in the LMPr group (3.005 ± 0.119 vs. 0.826 ± 0.126, 2.977 ± 0.161 vs. 0.902 ± 0.069) (*P* < 0.01). No statistically significant difference was found in their expression between the LMPr group and the control group (Figure 6).

## 4. Discussion

Lung protective ventilation has always been a very important measure in the treatment of ARDS [13]. Since the introduction of barotrauma and volume injury, the research of lung protective ventilation has been constantly innovated [14]. The energy-biological injury concept represented by the mechanical power has attracted more and more attention from the ICU medical doctors. At present, few studies are available on the VILI caused by the overload of mechanical power [15]. In the past, the establishment of the VILI model was mainly based on the volume injury caused by different tidal volumes or the pressure injury caused by different airway pressures [16]. Therefore, the strategy of small tidal volume ventilation and limited platform pressure ventilation was proposed. However, clinical studies revealed that VILI is still very common even if small tidal volumes and restrictive pressure ventilation are administered according to lung protection strategies [17]. Therefore, in this study, the VILI model caused by different levels of MP under constant volume control pressure was established. It was designed according to the simplified equation proposed by Gattinoni et al. [18], under the premise that the tidal volume, positive end-expiratory pressure, inspiratory flow, and other parameters are constant by changing the breathing frequency of the mechanical ventilation, in order to achieve the purpose of changing the size of MP. The results showed that the low respiratory rate reduced the degree of diffuse alveolar damage when subjected to low MP, and the damage included lung tissue inflammation, alveolar edema, or epithelial cell damage. When MP significantly increased after higher respiratory frequency, the diffuse alveolar damage was significantly aggravated, promoting the ultrastructural damage of the alveolar epithelium and endothelial cells and increasing the alveolar-capillary membrane permeability, with the increased recruitment of inflammatory cells [19]. Cytokines were released and participated in the systemic inflammatory response.

The chemokine CXCL10 plays an important role in a variety of diseases by binding to its receptor CXCR3 [20]. The expression of the three ligands of CXCR3 (CXCL9, CXCL10, and CXCL11) is significantly increased in various diseases such as interstitial cystitis, ulcerative colitis, and myositis [21], and previous studies found that blocking CXCL10 can reduce the degree of the damage of these diseases. Our previous research also found that CXCL10 gene expression is significantly increased in mechanically ventilated patients with ARDS [6], and this conclusion was also confirmed in this clinical study. The higher the MP, the higher the CXCL10 and CXCR3 expression. The animal experiments further confirmed that the levels of CXCL10 and CXCR3 in the serum of the rats with lung injury increased and were significantly and positively correlated with the level of MP. The above results indicated that the enhanced inflammatory response was induced by the action of the overloaded MP, which promoted the progression of VILI.

Previous studies [22] found that the activation of some chemokines enhances the migration of mast cells. Our research also showed that CXCL10 regulated the migration of mast cells to inflammatory sites, thereby affecting the pathophysiological process of VILI. CXCL10 and CXCR3 in the serum and tissues of the rats in the high MP group significantly increased compared with those in the control group. CXCL10 exerts its function by binding to its receptor CXCR3, which is a G protein-coupled receptor, which is expressed in a variety of cells such as lymphocytes and monocytes [23]. Recent studies showed that CXCR3 exists on mast cells in acute lung injury and chronic lung disease [24]. In addition, CXCL10 and CXCR3 continuously activate the oxidative burst and chemotaxis of mast cells in the form of an autocrine ring. Indeed, CXCL10 secreted by local tissues is one of the proteins involved in the recruitment of CXCR3-positive cells to the inflammatory area.

Mast cells may play an important role in the “energy-biological injury” of VILI caused by mechanical ventilation in patients with ARDS, although still few studies on this aspect are available. Mast cells are derived from different precursor cells in the bone marrow or other hematopoietic tissues [25] and are mainly distributed in the interface tissues, often on the mucosal surface between the body and the external environment [26]. When the mast cells receive the appropriate chemokine signals, they migrate to the skin, mucous membranes, airways, and other tissues and participate in the regulation of natural and adaptive immune responses. The excessive activation of mast cells is closely related to the occurrence and development of inflammatory diseases [25]. Mature mast cells express a variety of intra- and extramembrane receptors, which can in turn induce cell activation after binding to the corresponding ligands, leading to the release of various inflammatory mediators [27]. This study revealed that the chemotaxis of mast cells in the high MP lung injury group was significantly increased compared with the normal mechanically ventilated rats. Therefore, the occurrence of VILI might be significantly related to the recruitment of mast cells. During the mechanical ventilation treatment, the different mechanical stimulations of the airways and alveoli cause the mechanical stimulation of the mast cells, leading to their chemotaxis and degranulation, the release of inflammatory mediators, and the mediation of the energy-biological injury effect of VILI.

Although the middle and high MP mechanically ventilated groups showed an inflammatory response and release of inflammatory factors, the pathological results revealed no excessive alveolar expansion in the rat lung tissue. However, our research showed that the inflammatory response caused by MP under the high respiratory rate was more evident, and the pathological damage gradually aggravated with the increase of MP, with diffuse lung tissue damage after 24 hours of ventilation. The alveolar cavity was congested, and the edema was present, as well as interstitial; inflammatory cell infiltration, hyaline membrane formation, and more common alveolar collapse and alveolar epithelial and endothelial cell damage were observed [28]. Our results further highlighted the importance of preventing injuries induced by energy during mechanical ventilation. The monitoring of patients' MP should be emphasized even when protective mechanical ventilation is performed on ARDS patients. In other words, the overloaded MP is the main determinant of lung injury caused by the ventilator even when the tidal volume or transpulmonary driving pressure is within the “safe” range. It may also be due to improper settings or excessively strong spontaneous breathing, leading to a higher ventilation frequency, which may cause or aggravate lung damage [29]. Our results might also indirectly explain the reason why ARDS patients could not benefit from high-frequency oscillatory ventilation therapy in clinical practice. Although low tidal volume and restrictive drive pressure are used during high-frequency oscillatory ventilation, improperly high mechanical ventilation frequency leads to higher MP, which also causes or aggravates lung damage [30].

## 5. Limitations

This study contains some limitations. First, the patients were divided into the HMP group and LMP group according to whether the 24 h-mean-MP was higher or lower than 17.0 J/min. This threshold is based on previous literature reports and needs further verification. Second, the patient's CXCL10 and CXCR3 expression at 24 h was the only one evaluated, without dynamic monitoring of the trend of its change, while more time points need to be evaluated in future studies. Third, CXCL10/CXCR3 protein expression and gene expression were the two parameters measured in the injured lung tissues, and an in-depth analysis of the activation and inhibition of the signaling pathway they belong to was not performed. Furthermore, the morphological changes of the degranulation of mast cells under the electron microscope were not described. This, further research is needed.

## 6. Conclusions

The higher the overload of MP, the more severe the lung injury caused by the ventilator, and the higher the expression of CXCL10/CXCR3. Thus, CXCL10/CXCR3 might participate in the VILI energy-biological injury by mediating mast cell chemotaxis. This work might provide new targets and directions for VILI prevention and treatment.

## Figures and Tables

**Table 1:** Baseline levels of clinical and pathological characteristics in ARDS patients.

Index/group	HMPp group (*n* = 46)	LMPp group (*n* = 39)	*t*/*x*^2^/*Z*	*P*
Gender (M/F)	30/16	25/14	0.107	0.914
Age (years, *M* (QL, QU))	62 (47, 72)	61 (51, 69)	0.734	0.421
Weight (kg)	63.13 ± 9.43	64.41 ± 9.54	0.620	0.537
BMI (kg/m^2^)	25.12 ± 3.17	24.35 ± 4.33	0.944	0.347
SOFA	9.48 ± 2.63	8.61 ± 2.75	1.488	0.140
APACHE II	20.50 ± 6.12	21.02 ± 5.91	0.396	0.692
Tidal volume (mL)	370.68 ± 57.45	356.32 ± 49.25	1.225	0.224
PEEP (cmH_2_O)	12.24 ± 3.53	11.32 ± 3.91	1.140	0.257
Respiratory rate	28.96 ± 6.19	26.23 ± 5.28	2.166	0.033
Plateau pressure (cmH_2_O)	27.34 ± 4.23	25.37 ± 5.43	1.909	0.059
PaO_2_/FiO_2_ (mmHg)	129.56 ± 25.31	135.58 ± 31.43	0.978	0.331
Lactic acid (mmol/L)	6.65.±2.34	5.93 ± 2.51	1.367	0.175
Smoking history (Y/N)	18/26	15/24	0.227	0.820
Basic medical history (Y/N)	20/26	19/20	0.481	0.629

Data are mean ± standard deviation or number/total. ^∗^BMI: body mass index; SOFA: Sequential Organ Failure Assessment; APACHE II: Acute Physiology and Chronic Health Evaluation II; PEEP: positive end-expiratory pressure.

**Table 2:** CXCL10 and CXCR3 levels in the serum of the two groups of ARDS patients.

	LMPp group	HMPp group	*t*	*P*
CXCL10 (pg/mL)	1023.73 ± 237.81	1753.54 ± 329.89	11.513	<0.001
CXCR3 (pg/mL)	643.78 ± 143.02	1067.53 ± 271.73	8.759	<0.001

## Data Availability

The data used to support the findings of this study are available from the corresponding authors upon request.
